# The effectiveness of flow cytometric sorting of human sperm (MicroSort®) for influencing a child’s sex

**DOI:** 10.1186/1477-7827-12-106

**Published:** 2014-11-24

**Authors:** David S Karabinus, Donald P Marazzo, Harvey J Stern, Daniel A Potter, Chrispo I Opanga, Marisa L Cole, Lawrence A Johnson, Joseph D Schulman

**Affiliations:** Genetics & IVF Institute, 3015 Williams Dr, Fairfax, VA 22031 USA; Huntington Reproductive Center, 23961 Calle de la Magdalena, Suite 503, Laguna Hills, CA 92653 USA; 16920 Hardy Rd, Mount Airy, MD 21771 USA

**Keywords:** Human sperm, sperm sorting, Flow cytometry, Sex selection, IUI, IVF/ICSI, FET, ART procedures

## Abstract

**Background:**

Flow cytometric sorting can be used to separate sperm based on sex chromosome content. Differential fluorescence emitted by stained X- vs. Y-chromosome-bearing sperm enables sorting and collection of samples enriched in either X- or Y-bearing sperm for use to influence the likelihood that the offspring will be a particular sex. Herein we report the effectiveness of flow cytometric sorting of human sperm and its use in human ART procedures.

**Methods:**

This prospective, observational cohort study of the series of subjects treated with flow cytometrically sorted human sperm was conducted at investigational sites at two private reproductive centers. After meeting inclusion criteria, married couples (n = 4993) enrolled to reduce the likelihood of sex-linked or sex-limited disease in future children (n = 383) or to balance the sex ratio of their children (n = 4610). Fresh or frozen-thawed semen was processed and recovered sperm were stained with Hoechst 33342 and sorted by flow cytometry (n = 7718) to increase the percentage of X-bearing sperm (n = 5635) or Y-bearing sperm (n = 2083) in the sorted specimen. Sorted sperm were used for IUI (n = 4448) and IVF/ICSI (n = 2957). Measures of effectiveness were the percentage of X- and Y-bearing sperm in sorted samples, determined by fluorescence in situ hybridization, sex of babies born, IVF/ICSI fertilization- and cleavage rates, and IUI, IVF/ICSI, FET pregnancy rates and miscarriage rates.

**Results:**

Sorted specimens averaged 87.7 ± 5.0% X-bearing sperm after sorting for X and 74.3 ± 7.0% Y-bearing sperm after sorting for Y. Seventy-three percent of sorts were for girls. For babies born, 93.5% were females and 85.3% were males after sorting for X- and Y-bearing sperm, respectively. IUI, IVF/ICSI, and FET clinical pregnancy rates were 14.7%, 30.8%, and 32.1%, respectively; clinical miscarriage rates were 15.5%, 10.2%, and 12.7%.

**Conclusions:**

Flow cytometric sorting of human sperm shifted the X:Y sperm ratio. IUI, IVF/ICSI and FET outcomes were consistent with unimpaired sperm function. Results provide evidence supporting the effectiveness of flow cytometric sorting of human sperm for use as a preconception method of influencing a baby’s sex.

**Trial registration:**

NCT00865735 (ClinicalTrials.gov)

## Background

Human sperm sorted by flow cytometry can increase the likelihood that a child so conceived will be of a particular sex. This provides a preconception reproductive option for parents wishing to reduce sex-linked and sex-limited disease risk for their future children or to balance the sex ratio among their children. The intensity of the fluorescence emitted by the DNA of chromosomally normal, fluorescently stained sperm varies depending on the presence of the X- or the Y-chromosome. The X-chromosome contains more DNA than the Y-chromosome [1]; in humans, X-chromosome-bearing sperm have approximately 2.8% more total DNA than Y-bearing sperm [2, 3]. In sperm stained with a DNA-specific fluorochrome, this difference in DNA content is made evident by the intensity of the fluorescent signal emitted by the stained sperm, thereby allowing the differentiation of X- from Y-bearing sperm such that enriched populations of X- or Y-bearing sperm may be generated using flow cytometric sorting.

Improving the efficiency of food production was the impetus for the development of sex pre-selection in non-human mammals. Johnson and co-workers utilized the vital stain Hoechst 33342 (H33342) to stain the chromosomal DNA of X- and Y-bearing sperm and sorted the sperm nuclei into separate populations [4]. In subsequent experiments they stained and sorted living mammalian sperm to produce the first live births of rabbits and pigs with significantly skewed sex ratios [5, 6] followed by births of calves from live sorted sperm [7, 8]. In these animal studies [5–8] and another study [9] the offspring were all normal and showed no detrimental effect of sorting or from the use of the fluorescent stain. The first successful flow cytometric separation of X- and Y-bearing human sperm into enriched populations, the results of which were analyzed by fluorescence in situ hybridization (FISH), was subsequently undertaken by Johnson and multiple collaborators from Genetics & IVF Institute (GIVF) [2].

Flow cytometric sperm sorting was patented for mammalian applications by the United States Department of Agriculture (USDA; U.S. patent # 5,135,759). Because of GIVF’s extensive work with USDA scientist Lawrence Johnson on human applications of sperm sorting, coupled with our ability to undertake clinical work in this area, in 1992 USDA granted GIVF an exclusive license to apply the sperm sorting technology in humans. GIVF thereafter obtained USDA and IRB approval to initiate human clinical studies utilizing flow cytometric sperm sorting, at first for couples at risk for having children with sex-linked or sex-limited disease, and subsequently inclusive of family balancing. Sperm sorting was only available through enrollment and participation in the clinical study. GIVF has applied the registered trademark name MicroSort® (hereafter MicroSort) to the human sperm sorting process; the registered trademarks XSort® and YSort® (hereafter XSort and YSort, respectively) apply to sorting with MicroSort to increase the proportion of X-bearing sperm and Y-bearing sperm, respectively.

As described in the current report, MicroSort has been successfully employed in association with intrauterine insemination (IUI) and in vitro fertilization (IVF) with intracytoplasmic sperm injection (ICSI) to achieve numerous pregnancies, currently totaling over 1,300 live-born babies. Levinson et al. [10] reported the first human pregnancy resulting from MicroSort. Fugger et al. [11] reported the births of babies resulting from the use of sorted human sperm for IUI, IVF, or ICSI. Both fresh and frozen-thawed human sperm have been sorted to yield populations enriched in X-bearing or Y-bearing sperm [4, 12].

From the inception of human clinical application, GIVF had initiated and sponsored its own IRB-approved clinical trial of MicroSort. This had proceeded for several years, with accompanying reports of successful results both in the peer reviewed literature and in lay media. In 1999 the United States Food and Drug Administration (FDA) notified GIVF of its opinion that MicroSort should be classified as a medical device falling under FDA regulatory jurisdiction. GIVF responded that it believed MicroSort was an innovative medical method, rather than a medical device regulated by FDA, but the agency was unwilling to alter its opinion. GIVF therefore submitted to FDA an Investigational Device Exemption (IDE) application to study the safety and effectiveness of the MicroSort sperm separation technology. The IDE application was conditionally approved in May 2000 and received full approval in August 2001 to continue the investigation for both the Genetic Disease Prevention (GDP) and Family Balancing (FB) indications. From the beginning of the study, sorts were performed and sorted sperm were used under the supervision of physicians at GIVF in Fairfax, VA. In 2002, GIVF received FDA and IRB approval for a second investigational site which included a sorting laboratory. That site opened in Laguna Hills, CA, in 2003. The clinical study was concluded in March 2012, thereby ending MicroSort availability in the United States. MicroSort is currently offered by GIVF to patients in several other countries outside the United States.

In this paper we report the methods and overall effectiveness results from the MicroSort clinical study.

## Methods

The objectives of this prospective, observational cohort study, conducted under an FDA-approved IDE, were to determine the safety and effectiveness of flow cytometric sorting of human sperm. Only effectiveness results of the clinical study are presented in this report. Effectiveness was determined by measuring the ability of sorted sperm to increase the probability of conceiving an infant of the targeted sex. The two primary measures of effectiveness were fluorescence in situ hybridization (FISH) analysis of sorted sperm to determine the percentage of X- and Y-bearing sperm cells in sorted samples and the sex of babies born from the use of the sorted sperm. Secondary measures of effectiveness were pregnancy rates and the sex of prenatal fetuses (if prenatal sex determination was performed). Safety was determined by evaluating the rate of congenital malformations among infants born from the sorted sperm. The primary safety measure was the rate of major congenital malformations among infants born from sorted samples compared to that of the general population. Those results will be presented in a separate report. This study was conducted with Institutional Review Board approval (Chesapeake IRB; registration number IRB00000790) under an FDA-approved Investigational Device Exemption (IDE). The FDA clinical trials registration number was NCT00865735. The results reported here are from data collected between June 1994 and January 2012.

### Study population

The study population consisted of married couples who desired children of a specific sex to reduce the risk of sex-linked and sex-limited genetic disorders in their future children (Genetic Disease Prevention; GDP), or for balancing the sex ratio among their children (Family Balancing; FB). Sperm sorting with MicroSort was only available through enrollment and participation in the clinical study. Enrollment in the FB indication was limited to couples who had at least one child, desired to have a child of the under-represented sex among all of their children, and where the wife or the egg donor was younger than 40 years of age. Limitations on age or prior children did not apply to GDP participants. Both GDP and FB couples used donor sperm or oocytes if medically indicated.

### Subject selection

Participants were primarily fertile, married couples who met inclusion criteria, were enrolled in the study, and who sought reduced genetic disease risk or a balanced sex distribution among their children. The study enrollment also included couples undergoing treatment for infertility indications who qualified for and desired participation in the study. Table 1 contains the inclusion and exclusion criteria for the FB and GDP indications. Enrollment was conducted at the two investigational sites: at GIVF in Fairfax, VA, (1994–2012) and at the Huntington Reproductive Center (HRC) clinic in Laguna Hills, CA (2003–2012). Couples meeting inclusion criteria underwent clinical consultation, any indicated medical evaluation, and signed an informed consent form before being accepted as study participants. Documentation of genetic disease risk was reviewed by a medical geneticist on the study staff to confirm eligibility for enrollment and participation under the GDP indication. Once enrollment was complete, cycle management decisions, e.g., the use of IUI or IVF/ICSI, ovarian stimulation protocols, etc., were made between the participating couple and their physician.

**Table 1:** **Inclusion and exclusion criteria for participation in the MicroSort** ^**a**^ **clinical study: Genetic Disease Prevention (GDP) and Family Balancing (FB) indications** ^**b**^

Inclusion criteria	Criterion for GDP?	Criterion for FB?
The man and woman (couple) are married.	Yes	Yes
The couple combined must have at least one child (biological, adopted or stepchild).	No	Yes
The couple desires the under-represented gender among all of their children (biological, adopted or stepchild).	No	Yes
The couple wishes to minimize the risk of genetic disorders that are known or believed to be sex-linked or sex-limited.	Yes	No
The husband and wife, and donor and/or surrogate mother (if applicable) have negative laboratory test results for HIV-1 antibody, Hepatitis B surface antigen, and Hepatitis C antibody.	Yes	Yes
The wife or the donor of the eggs must be between the ages of 18–39 at the time of egg retrieval or insemination.	No	Yes
Both husband and wife agree to participate in the ongoing follow-up, as evidenced by providing signed medical release forms to obtain newborn and pediatric records for any children conceived during the clinical study.	Yes	Yes
Both husband and wife have signed an informed consent.	Yes	Yes
**Exclusion criteria**		
A history of a major congenital malformations or known chromosomal abnormality in the husband, wife or donor (egg or sperm) or in their prior children.	No	Yes
A clinically significant disease in the woman who will be carrying the pregnancy.	Yes	Yes
Abnormal, undiagnosed, gynecological bleeding in the woman who will be carrying the pregnancy.	Yes	Yes
Known allergy or hypersensitivity to the dye used for DNA staining in the woman who will be carrying the pregnancy.	Yes	Yes
Known current substance abuse in couple (husband and wife) that is the intended parents, or in the woman who will be carrying the pregnancy.	Yes	Yes

^a^MicroSort is a process of flow cytometric sorting human sperm to increase the proportion of X- or Y-chromosome-bearing cells in the sorted specimen. The differential fluorescence emitted by stained X- vs. Y-chromosome-bearing sperm enables the identification and selection of X- or Y-bearing cells such that the sorted specimen is enriched in the targeted sperm. Sorted sperm may be used to attempt to establish pregnancy so as to influence the likelihood that the baby will be of a particular sex.

^b^Indications = GDP, FB: Participation in the MicroSort clinical study to reduce the risk of sex-linked/ sex-limited genetic disease in future children (GDP) or to balance the sex ratio among current children (FB).

### IUI treatment cycles

Cycle monitoring for IUI cycles utilized either ovulation predictor kits or frequent transvaginal sonography coupled with serum progesterone, estradiol, and luteinizing hormone (LH) measurement, or some combination of ovulation monitoring tools. Gonadotropin stimulation was used, if indicated, after a discussion of additional risks related to multiple gestation and ovarian hyperstimulation syndrome. Insemination was performed 28–52 hr after detection of the LH surge, or 36–40 hr after human chorionic gonadotropin (hCG) administration. The lead follicle was 17–25 mm at the time of hCG administration, depending upon the stimulation protocol. Inseminations for IUI utilized only freshly sorted sperm and took place exclusively at either GIVF, the location of the investigational site and sperm sorting laboratory in Fairfax VA, or the investigational site at the HRC clinic, located in the same building as the sperm sorting laboratory in Laguna Hills, CA.

### IVF/ICSI treatment cycles

Participants undergoing IVF/ICSI treatment cycles underwent ovarian stimulation using various gonadotropin protocols that were in standard use at GIVF as well as at multiple national and international facilities of collaborating physicians. Freshly sorted sperm samples or cryopreserved sorted sperm samples were used for IVF/ICSI at the two investigational sites whereas only cryopreserved sorted specimens were used by the collaborating physicians. In both cases, the cryopreserved sorted sperm for IVF/ICSI were thawed and used without further processing.

### Sperm preparation and staining

Sperm preparation and sorting were performed at the sperm sorting laboratory at GIVF in Fairfax, VA, or at the sperm sorting laboratory in Laguna Hills, CA. Study participants provided either fresh or cryopreserved semen for sorting. Prior to evaluation and processing, freshly collected semen was allowed to liquefy at 35°C for 30 min; cryopreserved specimens were thawed according to instructions provided with the cryopreserved semen. All semen was evaluated for volume, concentration, percentage motile sperm, progression, and viability (eosin dye exclusion) before and after processing. Semen was processed by centrifugation through either glass wool columns or, after 1998, discontinuous density gradients (ISolate, 50%, 90%; Irvine Scientific, Santa Ana, CA). After processing, recovered sperm were washed and the sperm pellets re-suspended in medium [BWW (Irvine Scientific) supplemented with 10% bovine serum albumin (Sigma, St Louis, MO) before June, 2004, or either Ham’s F-10 or Sperm Washing Medium supplemented with 0.5% Human Serum Albumin (both Irvine Scientific) after June 2004]. Aliquots of 10 × 10^6^ sperm were then stained for 1 hr at 37°C with Hoechst 33342 (H33342; Calbiochem-Behring Corporation, La Jolla, CA) at a final concentration of 9 μM as previously described [2]. H33342 is a non-intercalating [13], membrane permeable [14, 15], DNA-specific fluorescent stain that binds non-covalently to poly-AT regions of the minor groove of the DNA helix [16]. It’s excitation and emission maxima are 350 nm and 456 nm, respectively [17].

After one hr of staining, each aliquot of stained sperm was sorted for one hr before being replaced by the next aliquot of freshly stained sperm. Sperm aliquots were stained sequentially and staining was timed so as to minimize the wait for the freshly stained aliquot after sorting the preceding aliquot was completed.

### Flow cytometric sperm sorting

Prepared, stained sperm were sorted as previously described [2]. Sperm were sorted using either a modified Epics® 753 (Coulter Corporation, Hialeah, FL) or modified FACS® Vantage flow cytometers (Becton-Dickinson Immunocytometry Systems, San Jose, CA) equipped with argon ion water cooled lasers (Coherent Inc., Santa Clara, CA). Instruments were modified according to [18]. Instruments were calibrated before each sort using H33342-stained sperm from a single human donor chosen because of the known, predictable performance of his sperm in response to the standardized staining and excitation conditions of sorting. Dulbecco’s phosphate buffered saline (Irvine Scientific) was used as sheath fluid. Fluorescence emitted by each stained sperm after UVA laser excitation (333–364 nm, 100 mW) was directed through a 400 nm long pass filter to forward (0°) and right angle (90°) detectors. Properly oriented sperm were identified and gated based on 90° fluorescence intensity. The sperm identified by the 90° gate were then gated on lower (YSort) or higher (XSort) 0° fluorescence intensity, and the sperm meeting the 0° fluorescence gating criteria were electrostatically deflected from the sample stream and into the collection container. For any given sort, only one type of sperm (X-bearing or Y-bearing) was intended for collection.

Sperm were analyzed at a rate of 3,000-3,500 cells per sec and the sorted sperm (predominantly X-bearing or predominantly Y-bearing) were collected at a rate of approximately 15–20 cells per sec into TYB Refrigeration Medium (Irvine Scientific) or other media. Thus, one hr of sorting could yield 60,000 to 80,000 sorted sperm. The actual number of sorted sperm collected per hr of sorting varied from specimen to specimen. IUI sorts were performed with a target of 200,000 motile sperm collected post-sort. Fresh IVF/ICSI sorts were performed with a target of 60,000 motile sperm collected post-sort. For specimens that were to be cryopreserved after sorting, the target was 100,000 motile sorted sperm post-thaw, based on test-freeze results obtained prior to sorting or, in the absence of those results, an assumed maximum post-thaw motility of 50% of the pre-freeze motility.

Post-sort sperm were centrifuged to concentrate recovered cells in a final volume of 400 μL for IUI, 200 μL for cryopreservation, or 60 μL for IVF/ICSI procedures in which freshly sorted sperm were to be used. Post-sort motility and progression were evaluated at 35°C under paraffin oil using Hoffman illumination. A sample from each of the sorted specimens was obtained and preserved for a post-sort quantitative determination of enrichment in X- or Y-bearing sperm (post-sort purity) using FISH. Sorted specimens were used fresh for IUI or IVF/ICSI at GIVF or HRC or were cryopreserved and stored at the laboratory for future IVF/ICSI use at GIVF, HRC, or for shipment to a collaborating physician’s facility.

The times required to perform the necessary steps in the preparation and sorting process were as follows: Liquefaction of raw semen - 30 min; evaluation and preparation for sorting - 1.5 hr; staining first sperm aliquot - 1 hr; sorting - up to 4 hr for IUI; evaluating sorted sperm and preparing sorted sperm for insemination - 45 min). To obtain the target number of sorted sperm for IUI or for post-sort cryopreservation, at least 4 aliquots of 10 × 10^6^ sperm each were prepared. Therefore, assuming a post-preparation recovery rate of 30%, the raw semen specimen for an IUI sort was expected to contain ≥140 × 10^6^ sperm at ≥50% motility. For an IVF/ICSI sort, raw semen containing 40–70 × 10^6^ sperm at ≥50% motility was expected to yield the 1–2 aliquots of 10 × 10^6^ prepared sperm for sorting. If an initial raw semen specimen did not contain the anticipated number of sperm, the husband was requested to produce additional semen specimens.

### Fluorescence in situ hybridization (FISH)

A sample containing approximately 5,000 sperm was taken from the sorted specimen for FISH evaluation of post-sort purity. The FISH procedure was a modification [19] of the one-DNA probe standard protocol (Vysis, Inc., Downers Grove, IL) as previously described [4] using alpha satellite DNA probes specific for the X and Y chromosomes. Briefly, sorted sperm were washed twice in PBS, air dried on a slide, fixed with 75% methyl alcohol-25% acetic acid, washed with 2X Saline Sodium Citrate (SSC; 0.3 M NaCl, 30 mM sodium citrate; Vysis, Inc.) at 37°C and allowed to air dry. The fixed, washed sperm were then treated with 50 mM dithiothreitol (DTT) in 0.1 M Tris–HCl (pH 8.0 at room temperature), washed with 2X SSC, and air-dried. Sperm were then concurrently denatured at 75°C and incubated with Vysis Spectrum CEP X orange/Y green probe mixture and Vysis Spectrum CEP Hybridization buffer (Vysis, Inc.) under a cover glass in a hybridization chamber. After the sperm DNA and the X- and Y-probe mixture hybridized, slides were washed with 0.4X SSC and counterstained with 4’,6-diamidino-2-phenylindole (DAPI; Vysis, Inc.). The labeled, counterstained sperm were evaluated at 600 X total magnification using an Olympus BX60 fluorescence microscope (Olympus America, Inc., Center Valley, PA) equipped with a dual band pass fluorescein isothiocyanate (FITC)/Rhodamine cube and DAPI filter. Sperm were initially identified using the DAPI filter then evaluated for presence of X- (red) or Y- (green) probe signal using the FITC/Rhodamine filter. At least 200 spermatozoa were counted for each patient sample. Samples were taken for FISH analysis after every sort, and results were successfully obtained on approximately 99% of the 7718 sorts performed.

### Post-sort specimen cryopreservation

For cryopreservation, sorted specimens were diluted 1:1 (v:v) with TEST Yolk Buffer Freezing Medium (Irvine Scientific), transferred to 1 mL Nunc cryotubes (Nunc, Kamstrup, DK) or 0.25 mL straws (IMV, Minneapolis, MN) and subsequently frozen in liquid nitrogen vapor using a programmable controlled rate freezer (Planar Kryo 10, TS Scientific, Perskie, PA). After vapor freezing, the sorted specimens were plunged into liquid nitrogen for storage until use. Frozen sorted specimens for IVF/ICSI were thawed at room temperature before use.

### Cycle outcomes and baby follow-up

Cycle outcome information was provided by physicians enrolled as collaborators in the clinical study. Because IUIs were only performed at the two investigational sites (Fairfax, VA, and HRC in Laguna Hills, CA), physicians at those sites provided IUI cycle outcome information in addition to outcome information for IVF/ICSI cycles in which freshly sorted sperm or cryopreserved sorted sperm were used. Collaborating physicians not at the Fairfax, VA, or the Laguna Hills, CA, sites only received cryopreserved sorted sperm for use in IVF/ICSI and agreed to provide the cycle outcome results. Cycle data were recorded on standardized clinical report forms (CRFs) which were then forwarded to GIVF for review by study personnel and data entry. Cycle data included medications used for ovarian stimulation, and retrieval, fertilization, cleavage, and PGD results. Other data included pregnancy testing results and results of any early ultrasounds performed to determine intrauterine localization and number of developing fetuses. A clinical pregnancy was defined as any pregnancy that had a sonographically-detected fetal sac with or without fetal heart activity, any miscarriage which occurred more than 35 days after insemination or embryo transfer, or any pregnancy with documented presence of fetal tissue. A clinical miscarriage was defined as the loss of a clinical pregnancy more than 35 days after insemination or embryo transfer, or any pregnancy loss which required a dilation and curettage.

Once clinical pregnancy was established it was customary that the female participant returned to the care of her OB/GYN for the duration of the pregnancy. It is possible that some collaborating physicians may have provided both ART treatment and obstetric care to their patients. Periodic follow-up calls were placed by study personnel to participants to obtain pregnancy status updates, including clinical miscarriages, pregnancy terminations, fetal reduction procedures, ectopic pregnancies, stillbirths, and other adverse events. In addition, the results of prenatal ultrasounds, including fetal sex determination, if performed (not required), chorionic villus sampling or amniocentesis were requested. Medical records were requested in order to identify, verify, evaluate and classify any events reported during follow-up calls. Data obtained from follow-up calls were recorded on CRFs by study personnel. A copy of the medical records containing results of the newborn physical examination performed at birth (birth records) and the pediatric evaluations performed by the baby’s physician throughout at least the first year of life (pediatric records) were requested from participants who had agreed, as part of the consenting process, to provide those medical records for babies born using MicroSort sperm. Each baby’s medical records (birth records and pediatric records) were independently reviewed by two board-certified medical geneticists (the study medical geneticists) engaged by the study sponsor to independently identify, evaluate and classify any congenital malformations and other adverse event results relating to the babies. These findings were recorded on CRFs by the study medical geneticists. Additional medical records and testing results were requested and reviewed as conditions dictated. A third medical geneticist was used to resolve any disagreement between the first two independent evaluations. Safety outcomes, including congenital malformation results and a more detailed description of the specific safety-related methods and findings, are not reported here and will be presented in a separate report.

### Data analysis

All data were recorded on CRFs which were submitted to study personnel for internal review for completeness. Completed CRFs were then sent to an independent data management firm for data entry into the clinical study’s database housed there. Periodic audits, edit checks, and reviews were performed on the database per the data management firm’s policies and procedures.

Although this clinical study was conducted to evaluate both the safety and the effectiveness of flow cytometric sorting of human sperm for subsequent use in ART procedures, only effectiveness results are presented in this report. However, it is necessary to make some mention of safety since the sample size was estimated taking into consideration both safety and effectiveness, with the larger sample size being selected.

The sample size for effectiveness was based on the FISH analysis of sorted sperm and the sex of babies born. For FISH analysis results, the objective was to demonstrate that the percentage of X-bearing sperm after XSort and the percentage of Y-bearing sperm after YSort was greater than 50%. For the sex of babies born, assuming the true success rate was at least 65%, 90 births provided 90% power to demonstrate that the success rate was greater than 50%.

The sample size for safety was based upon the rate of major congenital malformations in babies born, estimated to be 4% in the general population at the time the study was designed. The primary safety hypothesis was to demonstrate that the rate of major malformation was less than 6%, based on the assumption that the true major malformation rate was 4% plus a non-inferiority margin of 2 percentage points (4% + 2% = 6%). Thus, 1050 babies would provide 90% power to demonstrate non-inferiority. Because the sample size for major malformations was the largest, the study was powered based on a sample size of 1050 babies born.

Results are reported as means ± SD unless otherwise stated. Changes in clinical pregnancy rates, in clinical loss rates and percentages of babies having the targeted sex were tested by a test for trend, treating age groups as equally spaced, using the Cochrane-Mantel-Haenszel test [20] contained in SAS version 9.2 (The SAS Institute, Cary, NC). A *P* value <0.05 was considered to be significant.

## Results

### General

Between 1994 and 2012, 4993 couples were enrolled in the study; 7.7% (383/4993) for GDP and 92.2% (4610/4993) for FB. Overall, the mean age at enrollment was 38.5 ± 7.5 years for husbands and 35.5 ± 4.7 years for wives. For GDP, average husband and wife ages at enrollment were 35.2 ± 5.7 and 33.4 ± 4.3 years, respectively. For FB, the respective ages for husbands and wives at enrollment were 38.8 ± 7.5 and 35.6 ± 4.7 years. Of the 7718 sorts performed, 5635 (73.0%) were XSorts and 2083 (27.0%) were YSorts. 859 sorts (10.7%) were for GDP and 6859 (89.3%) for FB. Table 2 contains summary post-sort purity results for sorted sperm, and the sex of embryos, fetuses, and babies born from the use of sorted sperm. The sorted specimen contained an average of 87.8% (range 60.4-99.0; 95% CI 87.7-87.9) X-bearing sperm after XSorts and 74.3% (range 52.0-93.8; 95% CI 73.9-74.5) Y-bearing sperm after YSorts. Embryo sex results were in good agreement with post-sort FISH results while the fetal sex and baby sex results, though consistent with post-sort purity results, appeared elevated. An average of 215.7 × 10^6^ ± 166.9 × 10^6^ total motile sperm in raw semen yielded an average of 172.2 × 10^3^ ± 776.7 × 10^3^ motile sorted sperm available for use.

**Table 2:** **Post-sort purity** ^**a**^ **, embryo sex, fetus sex, and neonatal sex after flow cytometric sorting of human sperm**

	XSort^b^	YSort^b^
Sorted Sperm^c^	87.8 ± 5.0% X	74.3% ± 7.0 Y
	n = 5635	n = 2083
Embryo Sex^d^	87.0% **♀** ^e^	70.6% **♂** ^e^
	n = 3921	n = 3563
Fetus Sex^f^	90.5% **♀**	83.2% **♂**
	n = 567	n = 161
Baby Sex^g^	93.5% **♀**	85.4% **♂**
	n = 1010	n = 328

^a^Percentage of X- or Y-bearing sperm in the sorted sample after XSort and YSort, respectively.

^b^XSort, YSort: Sorting to recover X-bearing sperm or Y-bearing sperm, respectively.

^c^Determined by fluorescence in situ hybridization (FISH). Both values significantly different from 50% p < 0.001.

^d^Determined by embryo biopsy/preimplantation genetic diagnosis (PGD).

^e^♀, ♂ symbols denote female and male sex, respectively.

^f^ Determined by ultrasound, chorionic villus sampling, or amniocentesis. Includes cycles utilizing PGD.

^g^Determined by morphological examination at birth. Includes cycles utilizing PGD. Both values significantly different from 50% p < 0.001.

### Intrauterine insemination

4448 sorts were used in IUI cycles. 14.1% and 85.9% of sorts were for the GDP and FB indications, respectively; 80.4% of IUI sorts were XSorts and 19.6% were YSorts. Mean ages for husbands and wives undergoing IUI were 37.8 ± 7.2 and 35.1 ± 3.9 years, respectively. The overall IUI clinical pregnancy rate was 14.7% (653/4448) per cycle (Table  3), achieved with an average insemination dose of 217.1 × 10^3^ ± 71.7 × 10^3^ motile sperm. The majority of IUI cycles employed either no exogenous stimulation or clomiphene citrate alone for ovarian stimulation (data not shown). Clinical pregnancy rates per cycle decreased and miscarriage rates increased as female age increased (Table  3).

**Table 3:** **Pregnancy (PR) and spontaneous miscarriage (SAb) rates by female age for cycles in which flow cytometrically sorted human sperm were used for IUI**

Age^a^(yr)	Cycles (n)	Clinical pregnancies (n)	PR per cycle (%)^b^	SAb (n)	Clinical loss (%)^c^
<30	383	70	18.3	7	10.0
30-34	1614	264	16.4	31	11.7
35-39	2271	304	13.4	58	19.1
>39	180	15	8.3	5	33.3
All Cycles	4448	653	14.7	101	15.5

^a^Age at time of procedure.

^b^Statistically significant decrease with increasing age (p < 0.0001).

^c^Statistically significant increase with increasing age (p < 0.002).

### IVF/ICSI

A total of 2957 sorts were used in IVF/ICSI cycles. Of the IVF/ICSI sorts, 6.5% and 93.4% were for the GDP and FB indications, respectively; 59.1% were XSort and 40.9% were YSort. Mean ages for husbands and wives undertaking IVF/ICSI were 40.2 ± 6.7 and 35.1 ± 5.3 years, respectively. For IVF/ICSI sorts, the mean number of motile sperm after sorting was 118.5 × 10^3^ ± 1290 × 10^3^. Of the 41,008 oocytes retrieved, 32,586 were viable and appropriate for insemination. The overall fertilization rate was 71.4% (23,270/32,586). There were 22,283 two-pronucleate zygotes yielding 20,402 cleaved embryos for an overall cleavage rate of 91.6%. Overall, PGD was utilized in 37.5% of IVF/ICSI cycles; 34.0% of XSort IVF/ICSI cycles and 45.3% of YSort IVF/ICSI cycles employed the procedure. A mean of 2.4 ± 1.2 embryos (range 1–12; 95% CI 2.4-2.5) embryos were transferred per fresh cycle resulting in a mean IVF/ICSI clinical pregnancy rate of 30.8% (911/2957) per cycle (Table 4). The per-cycle clinical pregnancy rates for IVF/ICSI decreased with increasing egg source (wife or egg donor) age (Table 4); however, the apparent increase in miscarriage rates with age was not significant (p = 0.093; Table 4). There were 196 frozen embryo transfer (FET) cycles in which a mean of 3.1 ± 1.3 (range 1–7; 95% CI 2.7-3.5) frozen-thawed embryos were transferred, yielding 63 clinical pregnancies for a 32.1% per cycle FET clinical pregnancy rate. Among the FET clinical pregnancies, 8 miscarriages were reported for an FET clinical loss rate of 12.7%.

**Table 4:** **Pregnancy (PR) and spontaneous miscarriage (SAb) rates by female age for cycles in which flow cytometrically sorted human sperm were used for IVF/ICSI**

Age^a^ (yr)	Cycles (n)	Donor cycles (n)	Clinical pregnancies (n)	PR per cycle (%)^b^	SAb (n)	Clinical loss (%)
<30	334	173	146	43.7	11	7.5
30-34	822	46	278	33.8	23	8.3
35-39	1434	5	374	26.1	43	11.5
>39	163	5	31	19.0	4	12.9
Unconfirmed	204	201	82	40.2	12	14.6
All Cycles	2957	427	911	30.8	93	10.2

^a^Age of egg source at time of procedure (wife or egg donor, if used).

^b^Statistically significant decrease with increasing age (p < 0.0001), excluding unconfirmed age.

### Cycle outcomes and baby follow-up

A total of 1143 births with one or more babies resulted from 1627 clinical pregnancies. Table 5 contains a summary of the sex of babies born by ART type, sort type and female age (wife or egg donor, if used). There was no trend for the percentage of babies having the targeted sex to change with female age for any of the ART type-sort type subclasses (all p < 0.05). Of the 1358 babies born, 933 were from singleton pregnancies (68.7%), 410 from twin pregnancies (30.2%), and 15 from triplet pregnancies (1.1%). Sixteen ectopic pregnancies, 202 clinical spontaneous miscarriages and 24 selective reductions (6 for detected fetal abnormalities and 18 to reduce the risk of multifetal pregnancy) were reported. Of the 23 pregnancy terminations reported, 19 followed XSorts and 4 followed YSorts; 16 were for detected fetal abnormalities, 4 were for the non-targeted sex, 2 were unclassified and 1 was for a male fetus at risk for an X-linked disease. Of the babies whose sexes had been documented, 93.5% (944/1010) were of the targeted sex after XSorts and 85.4% (280/328) were the targeted sex after YSorts (Table 2). The rate of major congenital malformations for babies conceived with sorted sperm were statistically indistinguishable from general population controls (Marazzo DP., in preparation).

**Table 5:** **Babies born of the targeted sex** ^**a**^ **and of the not targeted sex** ^**b**^ **, by ART type** ^**c**^ **, sort type** ^**d**^ **and age** ^**e**^

ART^c^	Age^e^(yr)	XSort^d^		YSort^e^	
		# babies of targeted/not targeted sex	Targeted sex (%)	# babies of targeted/not targeted sex	Targeted sex (%)
**IUI**	<30	31/6	83.8	6/3	66.7
	30-34	162/12	93.1	27/4	87.1
	35-39	216/12	94.7	31/8	79.5
	>39	22/2	91.7	1/1	50.0
	All cycles	431/32	93.1	65/16	80.2
**IVF/ICSI** ^**f**^	<30	63/3	95.5	37/2	94.8
	30-34	144/9	94.1	67/11	85.9
	35-39	240/15	94.1	83/13	86.5
	>39	65/7	90.3	28/6	82.4
	Unconfirmed	1/0	100.0	0/0	0.0
	All Cycles	513/34	93.8	215/32	87.0

^a^Targeted sex = Female baby after XSort, Male baby after YSort.

^b^Not targeted sex = Male baby after XSort, Female baby after YSort.

^c^ART (assisted reproductive technology) type = intrauterine insemination (IUI) or in vitro fertilization/intracytoplasmic sperm injection (IVF/ICSI).

^d^Sort type = XSort, YSort: Sorting to recover X-bearing sperm or Y-bearing sperm, respectively.

^e^Age of egg source at time of procedure (wife or egg donor, if used).

^f^Includes FET cycles.

## Discussion

The results reported here show that the MicroSort sperm sorting resulted in a marked increase in the percentage of X- or Y-chromosome-bearing sperm in sorted specimens (Table 2). This is consistent with prior reports from GIVF [11, 21–23] and sorts analyzed independently by Vidal et al. [12]. The evaluation of many thousands of unsorted semen specimens utilizing FISH showed that the ratio of X- to Y-bearing sperm was invariably close to the expected 50:50 ratio (data not shown). Sorting caused a significant (p < 0.001) and biologically meaningful shift in the X:Y ratio to 88:12 after XSorts (n = 5635) and to 26:74 after YSorts (n = 2083). Those shifts equate, on average, to a 7.2-fold greater likelihood of a baby being female than male after an XSort, and a 2.9-fold greater likelihood of a baby being a male than a female after a YSort.

Successful sorting depends on the accurate detection of differences in fluorescent signal intensity between the X- and Y-bearing sperm. Strict adherence to standardized conditions of sperm preparation, staining, and instrument setup and operation minimize extrinsic effects on the fluorescent signal detection and thus, sort outcome. On the other hand, characteristics intrinsic to the sperm are more difficult to control. Variations in sperm head size, shape, and surface features (such as number, size and location of vacuoles) may affect the intensity of the fluorescent signal in ways similar to how those same characteristics affect light transmission through a lens. Variations in sperm chromatin packaging may affect stain uptake by limiting (or enhancing) stain access to DNA and impact sorting accuracy through decreased or increased signal intensity. While the intrinsic factors are more challenging to control and may be the greater contributors to sort-to-sort variation in sorting success, the results show that the vast majority of sorts resulted in a sorted specimen containing a high percentage of the targeted sperm.

The collection of sufficient sorted sperm for clinical application requires an adequate number of motile sperm in the raw semen. Not all participants were able to provide raw semen specimens of sufficient quality for sorting. Approximately 3% of sorts were cancelled for a variety of reasons, primarily related to semen quality: insufficient sperm numbers (<140 × 10^6^ sperm for IUI sorts and <70 × 10^6^ sperm for IVF/ICSI sorts) or motility (<50% motility) in the raw specimen; insufficient sperm recovered after processing, often due to extremely high degree of debris in the specimen; and/or poor sperm survival after processing. Although it was strongly recommended that study participants provide the results of a recent semen analysis prior to sorting, it was not required and not all did so. On the day of the sort, approximately 40% of participants provided a second semen specimen, and a few provided a third specimen, because the initial semen specimen was not of sufficient quality for sorting. If a sort was cancelled for reasons of poor semen quality, participants could reschedule a sort. In such cases, the quality of semen produced on the day of the rescheduled sort was adequate for sorting about half the time, suggesting that stress at the time of collection and/or inattention to the abstinence period may have been contributing factors to the poor initial specimen(s). Less than 1% of sorts were interrupted and/or cancelled due to cytometer or laser malfunctions; in these rare instances the sort was rescheduled after the malfunction had been addressed.

The ability of sperm sorting to increase the percentage of X-bearing sperm in the sorted specimen could be of benefit to couples wishing to avoid having children affected by sex-linked disease. There are over 1,100 X-linked diseases and approximately 60 Y-linked diseases [24]. Due to the fact that females have two X chromosomes (one of which undergoes X-inactivation), it is primarily the male child that is affected. This makes the greater effectiveness of sorting for X-bearing sperm particularly useful for helping reduce the likelihood of conceiving a child affected by the disease. In cases of classical X-linked disorders, sorting for X-bearing sperm would increase the likelihood of conceiving a girl to approximately 90% and decrease the likelihood of conceiving an affected male child from 25% to 2.5%.

The embryonic sex data (as determined by PGD) show proportions of XX embryos after XSort and XY embryos after YSort were consistent with the post-sort FISH results. However, the prenatal sex distributions for fetuses, determined in those who underwent ultrasound, CVS or amniocentesis for sex identification, and the sex of babies born, as determined by physical exam at birth, while paralleling the predicted outcomes, appeared increased (Table 2). Closer examination showed the sex distributions for babies born for XSort IUIs, YSort IUIs, XSort IVF/ICSIs, and YSort IVF/ICSIs (Table 5) were 5.3, 5.9, 6.0, and 12.7 percentage points greater, respectively, than the respective overall post-sort FISH results shown in Table 2. An imperfect agreement between the sex distribution of babies born and the post-sort FISH results is not unexpected and we speculate that for X-and YSort IUIs and XSort IVF/ICSIs, the 5 to 6 percentage point difference between values for those two variables, because of their uniformity across sort types and ART types, likely reflects normal variation (noise) characteristic of these data. However, the two-fold greater difference between the sex distribution of babies born and the post-sort FISH results for YSort IVF/ICSIs, which equates to 17 more male babies born than would have resulted if the percentage point difference between the baby sex distribution and the post-sort FISH results had been 5–6 percentage points, suggests something other than systemic noise. We suggest that the utilization of PGD in a greater percentage of YSort vs. XSort IVF/ICSI cycles (45% vs. 34%) potentially contributed to the higher than expected rate of male births after YSort IVF/ICSIs. Because PGD is very robust for identifying embryonic sex, the utilization of PGD in YSort IVF/ICSI cycles would be expected to yield a higher rate of male embryos identified and transferred, and ultimately result in an elevated rate of male births. Another potential contributor to the higher than expected rate of male births after YSort IVF/ICSIs could have been unreported miscarriages or terminations, resulting in the greater than expected survival of male vs. female pregnancies. It is also possible that YSorts, in addition to selecting Y-bearing sperm, also selected some X-bearing sperm of impaired competence possibly arising from the effects of passage through the flow cytometer or some unknown selection mechanism during the sorting. This could have increased the effective percentage of functional Y-bearing sperm in the sorted IVF/ICSI specimen and thereby the rate of male fetuses and babies. However, if this had occurred, one would expect this to also have been reflected in the sex distribution results for YSort IUIs, which it was not. It should be noted that determination of embryonic sex and prenatal fetal sex by any method was not required of participants, and therefore was not performed for all participants. Because PGD results were reported for approximately 40% of IVF/ICSI cycles and results of fetal sex determinations were reported for approximately 50% of fetuses, caution should be taken in the interpretation of, and speculation regarding the reasons for, the apparent differences between post-sort purity and baby sex distribution.

In addition to increasing the proportion of X- or Y-bearing sperm in the sorted sample, the current results indicate that the function of flow cytometrically sorted human sperm was not adversely affected. The IUI results (Table 3) show the pregnancy rates resulting from the use of sorted sperm were comparable to rates published in the literature. Published IUI pregnancy rates are generally 10-15% per cycle [25–27], whereas the overall IUI pregnancy rate in the current study was 14.7% in a population of participants presumed to have normal fertility potential. Theoretically, the presumed normal fertility status of most of the current study’s participants could have potentially increased the IUI pregnancy rate over that reported for studies that did not employ sorted sperm. Because the majority of the current study’s participants were enrolled under the FB indication, which required at least one previous child, and some participants that were enrolled under the GDP indication also had one or more children, primary infertility was likely not a factor. Similarly, because of the sperm requirements for sorting, male factor infertility was likely not a factor. On the other hand, the relatively low numbers of sorted sperm available for insemination, coupled with the mean age of the wife at insemination (35.1 ± 3.9 years) could be anticipated to lower the IUI pregnancy rate.

The number of motile sorted sperm inseminated in the current study (0.217 × 10^6^) was considerably lower than the range of threshold motile sperm doses (0.8 to >20 × 10^6^) reported by Ombelet et al. [28] in their review of sperm traits predictive of IUI outcomes. It was also lower than their proposed motile sperm threshold dose of >1.0 × 10^6^, above which IUI success was expected to be significantly improved. While doubling the inseminated dose of motile sorted sperm could possibly have resulted in an increased IUI pregnancy rate in the current study, in most cases the sperm dose would still have been lower than the threshold values discussed above. The detrimental effects of increased sorting time on sperm longevity would likely have had some counterbalancing effect on whatever benefit might have been derived from the increased number of sorted sperm that were obtained by increasing sorting time. However, the insemination of very low sperm numbers has been reported to result in pregnancies [29–33], indicating factors other than motile sperm numbers impact IUI pregnancy rate. The many factors that can impact IUI success and the different combinations of those factors among patient populations, coupled with the variation among physicians in the methods utilized for infertility treatment, likely can result in practice to practice variability in IUI results. Nonetheless, reports from multiple large studies show that overall IUI pregnancy rates fall between 10% and 15% per cycle [33–40].

The relatively low number of sorted sperm available for insemination was due largely to attrition during the multiple processing steps and the small proportion of properly oriented sperm passing through the flow cytometer during sorting. It was also due, in part, to the balancing of prolonged sort times to maximize sperm recovery against optimizing sperm longevity by minimizing the amount of time between semen collection and insemination. These multiple factors resulted in 0.6% to 1.0% of total sperm being recoverable for use. Given these factors, a low IUI pregnancy rate with sorted sperm would be expected if the sorting process adversely affected sperm function, particularly considering the sperm dose inseminated. On the contrary, our current results show IUI per cycle pregnancy rates to be consistent with other reports in the literature.

Results from IVF/ICSI cycles in the current study are consistent with published values for fertilization rate [41–44], cleavage rate [41, 45–47], and pregnancy rate [48] and provide additional information regarding the effect of sorting on sperm function. If sorting did adversely affect sperm function, one would expect lower rates of fertilization, cleavage and pregnancy, which was not the case. Furthermore, the spontaneous miscarriage rates for pregnancies achieved using sorted sperm (Table 3, Table 4) were comparable with those reported for the general population [40] and for IUI [49–51] and IVF/ICSI [50, 51] indicating that sorting did not adversely impact post-implantation, first trimester fetal development. Combined, the IUI and IVF/ICSI results indicate that sorted sperm were capable of fertilization in vivo and in vitro, and the use of sorted sperm did not appear to interfere with normal embryonic development and resulted in pregnancies at rates comparable to those seen when unsorted sperm are utilized. Furthermore, the FET results are consistent with literature reports in terms of clinical pregnancy rates [52–55] and miscarriage rates [52, 53, 55], indicating that frozen embryos arising from the use of sorted sperm were able to effect and maintain pregnancy at rates similar to those for frozen embryos resulting from the use of unsorted sperm.

It is notable that, overall, XSorts were the predominant sort type requested by participants, being performed nearly 3 times more often than YSorts (5635 XSorts vs. 2083 YSorts = 2.7 to 1). A combination of reasons may explain the more common preference for female babies among the participants in this study; these include 1) the greater likelihood of the desired sex outcome given the higher mean percentage of X-bearing sperm after XSorts relative to YSorts (88% versus 74%), 2) an overall parental or cultural preference for females in the FB patient population, and 3) a contribution of genetic disease prevention (GDP) to this preference. When the XSorts and YSorts for GDP were subtracted from their respective totals, the remaining ratio was still skewed toward a female preference (4813 XSorts vs. 2046 YSorts = 2.4 to 1).

## Conclusions

Flow cytometric sorting of human sperm with MicroSort resulted in a biologically meaningful shift in the expected 50:50 ratio of X- to Y-bearing sperm found in normal ejaculated semen. The use of sorted sperm (MicroSort) increased the chances of conceiving a child of a targeted sex. The sorted sperm yielded IUI, IVF/ICSI and FET outcomes consistent with outcomes reported in the literature using unsorted sperm, indicating that sperm function was unimpaired. These results illustrate the effectiveness of flow cytometric sorting of human sperm for subsequent use in ART as a preconception option for families wishing to reduce the risk of genetic disease or to balance the sex distribution among their children.

## Abbreviations

ART: Assisted reproduction technologies
BA: Bovine serum albumen
BWW: Biggers-Whitten-Whittingham medium
CA: California
DAPI: 4’,6-diamidino-2-phenylindole
CRF: Clinical report form
DNA: Deoxyribonucleic acid
FB: Family balancing
FDA: United States Food and Drug Administration
FET: Frozen embryo transfer
FISH: Fluorscence in situ hybridization
FITC: Fluorescein isothiocyanate
FL: Florida
GDP: Genetic disease prevention
GIVF: Genetics & IVF Institute
H33342: Hoechst 33342
hCG: Human chorionic gonadotropin
HRC: Huntington Reproduction Center
IUI: Intrauterine insemination
IDE: Investigation device exemption
IRB: Institutional review board
IVF/ICSI: In vitro fertilization with intracytoplasmic sperm injection
LH: Luteinizing hormone
MO: Missouri
PGD: Preimplantation genetic diagnosis
SD: Standard deviation
USDA: United States Department of Agriculture
UVA: Ultraviolet A
VA: Virginia.
